# Identification of SNPs and Candidate Genes for Milk Production Ability in Yorkshire Pigs

**DOI:** 10.3389/fgene.2021.724533

**Published:** 2021-10-05

**Authors:** Lijun Shi, Yang Li, Qian Liu, Longchao Zhang, Ligang Wang, Xin Liu, Hongmei Gao, Xinhua Hou, Fuping Zhao, Hua Yan, Lixian Wang

**Affiliations:** Institute of Animal Science, Chinese Academy of Agricultural Sciences, Beijing, China

**Keywords:** Yorkshire pig, litter weight gain, GWAS, SNP, candidate gene

## Abstract

Sow milk production ability is an important limiting factor impacting suboptimal growth and the survival of piglets. Through pig genetic improvement, litter sizes have been increased. Larger litters need more suckling mammary glands, which results in increased milk from the lactating sow. Hence, there is much significance to exploring sow lactation performance. For milk production ability, it is not practical to directly measure the milk yield, we used litter weight gain (LWG) throughout sow lactation as an indicator. In this study, we estimated the heritability of LWG, namely, 0.18 ± 0.07. We then performed a GWAS, and detected seven significant SNPs, namely, *Sus scrofa* Chromosome (SSC) 2: ASGA0010040 (*p* = 7.73E-11); SSC2:MARC0029355 (*p* = 1.30E-08), SSC6: WU_10.2_6_65751151 (*p* = 1.32E-10), SSC7: MARC0058875 (*p* = 4.99E-09), SSC10: WU_10.2_10_49571394 (*p* = 6.79E-08), SSC11: M1GA0014659 (*p* = 1.19E-07), and SSC15: MARC0042106 (*p* = 1.16E-07). We performed the distribution of phenotypes corresponding to the genotypes of seven significant SNPs and showed that ASGA0010040, MARC0029355, MARC0058875, WU_10.2_10_49571394, M1GA0014659, and MARC0042106 had extreme phenotypic values that corresponded to the homozygous genotypes, while the intermediate values corresponded to the heterozygous genotypes. We screened for flanking regions ± 200 kb nearby the seven significant SNPs, and identified 38 genes in total. Among them, 28 of the candidates were involved in lactose metabolism, colostrum immunity, milk protein, and milk fat by functional enrichment analysis. Through the combined analysis between 28 candidate genes and transcriptome data of the sow mammary gland, we found nine commons (ANO3, MUC15, DISP3, FBXO6, CLCN6, HLA-DRA, SLA-DRB1, SLA-DQB1, and SLA-DQA1). Furthermore, by comparing the chromosome positions of the candidate genes with the quantitative trait locus (QTLs) as previously reported, a total of 17 genes were found to be within 0.86–94.02 Mb of the reported QTLs for sow milk production ability, in which, NAV2 was found to be located with 0.86 Mb of the QTL region ssc2: 40936355. In conclusion, we identified seven significant SNPs located on SSC2, 6, 7, 10, 11, and 15, and propose 28 candidate genes for the ability to produce milk in Yorkshire pigs, 10 of which were key candidates.

## Introduction

The mammary gland is a ubiquitous morphological feature of mammals, and lactation is an essential process in mammalian reproduction, including the secretion of milk from mammary glands. For offspring, depending on milk is a key strategy to the life history of all mammals. During lactation, maintaining body growth and milk production for the dam is necessary, thus energy requirement is high. In the past few decades, genetic and management changes have occurred, and the modern sow is subject to additional challenges. Litter size is one of the most important factors affecting milk production in a sow (Eissen et al., 2000), and piglet survival after birth is negatively affected by increasing litter size (Wang et al., 2017). During this period, the litter size of pigs has increased and will continue as an important goal trait in pig breeding programs around the world (Spötter and Distl, 2006; Baxter et al., 2013). In general, larger litters need more suckle mammary glands, which results in increased milk from the lactating sow (Auldist et al., 1998). The survival of offspring can be enhanced by milk yield, which satisfies the immunological needs of offspring and assists in the endocrine maturation of neonates (Goldman, 2002). In response to greater suckling intensity, sows have to produce more milk to nurse more piglets (Auldist et al., 1998; Revell et al., 1998). Additionally, poor lactation traits lead to early culling, which affects the profitability of commercial producers. Hence, it is of economic importance to improve lactation performance in pigs, and it is necessary to include lactation traits in the breeding goals.

The genetic improvement of sow lactation performance is hindered due to the difficulty of collecting accurate phenotypes. Unlike dairy cattle, it is not possible to directly measure the sow milk yield. Different experimental methods have been proposed to measure pig milk production ability, such as the isotope dilution method (Pettigrew et al., 1987) and the weighsuckle-weigh method (Elsley, 1971). These methods are expensive, complicated, and labor-intensive, and are difficult to be implemented on a routine basis in a commercial herd. A simpler and more straightforward measurement for an increase in body weight of piglets during lactation has been reported and is considered as an indicator trait for milk production ability (Revell et al., 1998; Bergsma et al., 2008). In 2016, DM. Thekkoot et al. estimated the heritability of litter weight gain (LWG) as an indicator of lactation trait in Yorkshire and Landrace sows, namely 0.16–0.22 and 0.12–0.20, respectively (Thekkoot et al., 2016a).

A Genome-Wide Association Study (GWAS) is an effective strategy to examine the underlying genetics of complex traits (Goddard and Hayes, 2009). Many studies have identified candidate markers associated with important economic traits in pigs, such as meat quality (Falker-Gieske et al., 2019) and growth (Zhang et al., 2019). For LWG traits in Yorkshire sow lactation, the GWAS detects two quantitative trait locus (QTLs) on *Sus scrofa* Chromosome (SSC) 7 (126 and 101 Mb) (Thekkoot et al., 2016b).

Until now, there has been little known about the heritability and genomic prediction of sow milk production ability. In this study, we aimed to estimate the heritability of LWG of the sow during lactation, to perform a GWAS for proposing the single nucleotide polymorphisms (SNPs) and candidate genes, and to conduct the combined analysis with the reported swine mammary gland transcriptome data and GWAS data for further insights into the candidates involved in sow milk synthesis.

## Materials and Methods

### Animals and Phenotypic Data

In this study, a total of 985 Yorkshire sows involved in 96 sire families, were recorded between 2019 and 2020 in Shanxi and Liaoning Province, China. These sows were fed with the fodders prescribed by their farms, in which, the regular quarantine inspection was carried out. For each sow, only one production record was performed, and 985 individuals were involved in 1–8 parity.

As it was not practical to directly measure the milk production ability of sows, our study weighed all non-mummified piglets at birth, death, weaning, and at the time of fostering. This allowed us to quantify the exact weight gain of each piglet for each sow. We calculated the LWG for each sow by summing up the increase in weight of all piglets nursed by that sow and considered it as a potential indicator for milk production ability. The formula for calculating LWG was as follow:

 LWG (kg)= litter weight at weaning−litter weight at birth −litter weight at the time of fostering in +litter weight at death+litter weight at the time of fostering out

### Genetic Parameters Estimation for LWG

We estimated the genetic parameters of LWG with an animal model. The genetic parameters and estimated breeding values (EBV) were performed by the ASReml package as the following model:

y=Xb+Za+e

where y is a vector of phenotypic records (LWG of the sow); b is a vector of fixed effects containing herd by farm and production batch (nine levels), parity (five levels: 1, 2, 3, 4, and 5–8), and days of lactation (three levels: ≤ 18, 19–21, and > 21); X is a design matrix that associates b with y ; a is the vector of additive genetic effects; Z is the corresponding incidence matrix , and e is the vector of random residual effects. Variances of random effects are defined as $\text{V}\left(\text{a}\right)=G\sigma_{a}^{2}$ for the polygenes and $\text{V}\left(\text{e}\right)=I\sigma_{e}^{2}$ for the residuals, where the G is the additive genetic relationship matrix, I is the identity matrix, V(a) is the additive genetic variance, and V(e) is the residual variance. In this study, 985 sows were traced back to four-generation pedigrees to construct the kinship matrix, and a total of 2,415 individuals were included.

### Genotyping and Quality Control

Ear samples of the 985 Yorkshire sows were collected in farms. For each ear, DNA was isolated with a commercially available kit, Q1Aamp DNA Mini Kit (QIAGEN, Germany). In total, 985 sows were then genotyped with the GenSeek Genomic Profiler (GGP) Porcine 50K (50,697 SNPs, Illumina, San Diego, CA, United States).

With PLINK (Purcell et al., 2007), we removed the SNPs with minor allele frequencies < 0.01, and a deviation from Hardy-Weinberg equilibrium (HWF) *p* values < 0.001. A dataset containing 36,871 SNPs and 985 animals was used for further analysis. All SNP positions were annotated based on pig genome assembly *Sscrofa* 11.1. The genotype data used for GWAS was submitted to public repositories, and the DOI was 10.6084/m9. figshare.16545915 (https://figshare.com/s/edda38a1c99aa7ab7ae0).

### Genome-Wide Association Study

We utilized the EBV of LWG as the dependent variable to perform GWAS by Fixed and random effect model Circulating Probability Unification (FarmCPU). FarmCPU is a multi-locus model that incorporates multiple markers simultaneously as covariates to partially remove the confounding effect between testing markers and kinship (Liu et al., 2016). A genome-wide Bonferroni correction threshold of 0.05/36,871 (i.e., 1.36E-06) was implemented to correct for multiple testing and assess the significance level for each SNP. The Manhattan and quantile-quantile (Q-Q) plots were drawn by R packages (http://cran.r-project.org/web/packages/gap/index.html).

In addition, we estimated the least square mean of sow LWG phenotypes for homozygous and heterozygous genotypes of the seven significant SNPs with standard error (SE) by SAS9.2 (SAS Institute, Cary, NC, United States).

### Gene Contents and Functional Annotation

We used the BioMart in Ensembl database to retrieve candidate genes within 200 kb (Zhao et al., 2011) of significant SNPs based on the pig reference genome (Sscrofa11.1). To provide insight into the functional enrichment of candidate genes identified in this study, we performed gene ontology (GO) and Kyoto Encyclopedia of Genes and Genomes (KEGG) analysis with the KOBAS (http://kobas.cbi.pku.edu.cn/kobas3/genelist/) (Xie et al., 2011).

### Combined Analysis With the Reported Transcriptome and GWAS Data

To further confirm the key candidates, we performed the combined analysis between the results of this study and reported transcriptome research of the sow mammary gland (Palombo et al., 2018).

Based on the gene location information in the Ensembl database (http://asia.ensembl.org/index.html) and reported GWAS, it was considered that the candidate genes located within 5 Mb to the peak of QTLs in the previous GWAS were promising candidates associated with the ability to produce milk.

## Results

### Descriptive Statistics and Heritability of LWG Trait

**FIGURE 1:**
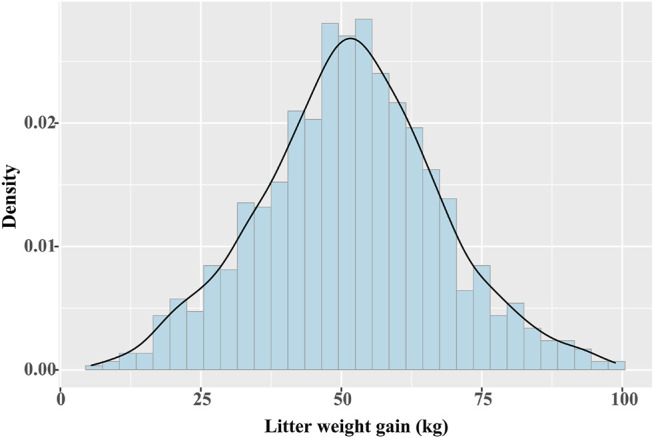
Distribution of milk production ability for 985 Yorkshire pigs.

For 985 Yorkshire pigs, the average days of lactation were 19.13. We calculated the descriptive statistics of LWG throughout lactation: number sows ∼ 985, mean ∼ 51.65 kg, standard deviation ∼ 16.05, maximum ∼ 98.74 kg, and minimum ∼ 5.54 kg. Figure 1 shows the distribution of LWG, which indicated the data was normal.

We estimated the heritability of sow LWG: 0.18 ± 0.07, in which, the estimated additive variance was V(a) = 25.95 ± 10.85, and residual variance was V(e) = 119.69 ± 10.30. Furthermore, we estimated the breeding value and include the results in Supplementary Table S1.

### GWAS and Identification of Candidate Genes

**FIGURE 2:**
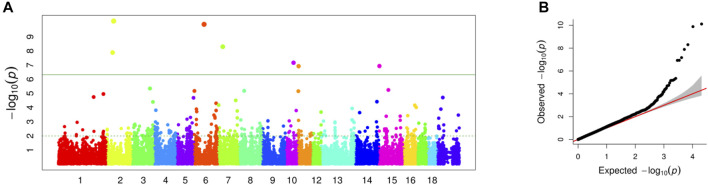
**(A)** Manhattan plot of association results for sow milk production ability. The green line indicated *p* = 1.36E-06. **(B)** Quantile-quantile plot of 36,871 SNPs in genome-wide association study for milk production ability.

In this study, a total of 985 sows with the EBVs of LWG and genotypes were used for the GWAS by FarmCPU. The Manhattan and Q-Q plots are shown in Figures 2A,B, respectively. Seven genome-wide significant SNPs were identified: ASGA0010040 (*p* = 7.73E-11) and MARC0029355 (*p* = 1.30E-08) located on SSC 2, WU_10.2_6_65751151 (*p* = 1.32E-10) located on SSC6, MARC0058875 (*p* = 4.99E-09) located on SSC7, WU_10.2_10_49571394 (*p* = 6.79E-08) located on SSC10, M1GA0014659 (*p* = 1.19E-07) located on SSC11, and MARC0042106 (*p* = 1.16E-07) located on SSC15 (Table 1).

**TABLE 1:** Candidate genes associated with milk production ability by genome-wide association study

SNP name	SSC	Position	*p*-value	SNP effect	Candidate genes	Gene symbol	Gene full name	Distance (kb)
ASGA0010040	2	39768974	7.73E-11	0.58	ENSSSCG00000013351	NAV2	Neuron navigator 2	Within
MARC0029355	2	33568298	1.3E-08	−0.71	ENSSSCG00000013338	SLC5A12	Solute carrier family 5 member 12	Within
					ENSSSCG00000013339	ANO3	Anoctamin 3	49.69
					ENSSSCG00000013340	MUC15	Mucin 15, cell surface associated	118.04
WU_10.2_6_65751151	6	71788235	1.32E-10	−0.78	ENSSSCG00000003417	DISP3	Dispatched RND transporter family member 3	147.29
					ENSSSCG00000025667	FBXO2	F-box protein 2	38.80
					ENSSSCG00000003421	FBXO6	F-box protein 6	14.01
					ENSSSCG00000003419	MAD2L2	Mitotic arrest deficient 2 like 2	9.05
					ENSSSCG00000003423	DRAXIN	Dorsal inhibitory axon guidance protein	Within
					ENSSSCG00000022401	AGTRAP	Angiotensin II receptor associated protein	37.13
					ENSSSCG00000046656	NA	NA	57.69
					ENSSSCG00000003428	MTHFR	Methylenetetrahydrofolate reductase	75.40
					ENSSSCG00000003429	CLCN6	Chloride voltage-gated channel 6	93.74
					ENSSSCG00000003431	NPPB	Natriuretic peptide B	131.26
					ENSSSCG00000003432	KIAA2013	KIAA2013	187.31
					ENSSSCG00000028965	U5	SnRNA	182.81
MARC0058875	7	24865378	4.99E-09	−0.57	ENSSSCG00000030874	NA	NA	153.33
					ENSSSCG00000033414	NA	NA	105.83
					ENSSSCG00000027921	NA	NA	72.79
					ENSSSCG00000001447	NA	NA	75.19
					ENSSSCG00000025071	BTNL2	Butyrophilin like 2	55.52
					ENSSSCG00000001453	HLA-DRA	SLA-DRA:MHC class II DR-alpha	30.22
					ENSSSCG00000001455	SLA-DRB1	MHC class II histocompatibility antigen SLA-DRB1	16.40
					ENSSSCG00000001457	SLA-DQB1	SLA-DQ beta1 domain	77.73
					ENSSSCG00000001456	SLA-DQA1	MHC class II histocompatibility antigen SLA-DQA	86.12
					ENSSSCG00000001459	HLA-DOB	SLA-DOB:MHC class II, DO beta	165.06
					ENSSSCG00000025593	TAP2	Transporter 2, ATP binding cassette subfamily B member	178.34
					ENSSSCG00000026951	PSMB8	Proteasome 20S subunit beta 8	187.75
					ENSSSCG00000001463	PSMB9	Proteasome 20S subunit beta 9	190.22
					ENSSSCG00000025618	TAP1	Transporter 1, ATP binding cassette subfamily B member	197.40
WU_10.2_10_49571394	10	44833617	6.79E-08	0.76	ENSSSCG00000011040	CACNB2	Calcium voltage-gated channel auxiliary subunit beta 2	Within
					ENSSSCG00000011041	NSUN6	Putative methyltransferase NSUN6	48.83
					ENSSSCG00000040106	NA	NA	155.91
M1GA0014659	11	4191013	1.19E-07	0.36	ENSSSCG00000024064	RNF6	Ring finger protein 6	158.85
					ENSSSCG00000009298	CDK8	Cyclin dependent kinase 8	9.92
					ENSSSCG00000009300	WASF3	WASP family member 3	70.16
MARC0042106	15	3073986	1.16E-07	0.43	ENSSSCG00000015677	LYPD6B	LY6/PLAUR domain containing 6B	101.07
					ENSSSCG00000022919	KIF5C	Kinesin family member 5C	116.36

Note: SSC: *Sus scrofa* Chromosome; NA: indicates novel gene in Ensembl database.

**FIGURE 3:**
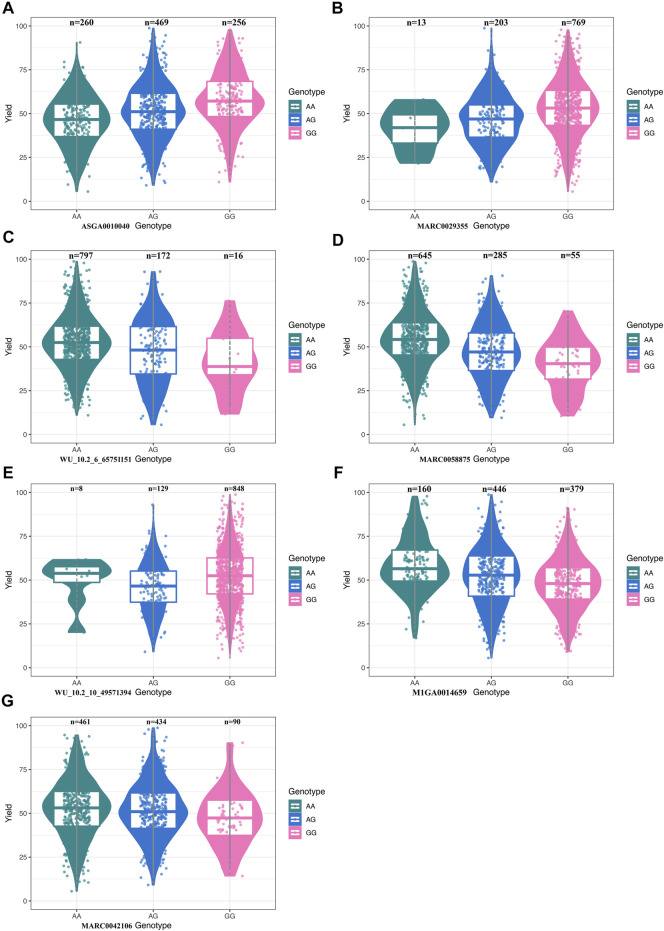
**(A)** Boxplot for litter weight gain (LWG) and the genotype at SNP ASGA0010040. **(B)** Boxplot for LWG and the genotype at SNP MARC0029355. **(C)** Boxplot for LWG and the genotype at SNP WU_10.2_6_65751151. **(D)** Boxplot for LWG and the genotype at SNP MARC0058875. **(E)** Boxplot for LWG and the genotype at SNP WU_10.2_10_49571394. **(F)** Boxplot for LWG and the genotype at SNP M1GA0014659. **(G)** Boxplot for LWG and the genotype at SNP MARC0042106.

We performed the distribution of phenotypes for LWG by the genotype of the significant SNPs, the results of which can be seen in Figure 3. These data of ASGA0010040, MARC0029355, MARC0058875, WU_10.2_10_49571394, M1GA0014659, and MARC0042106 showed that the extreme phenotypic values corresponded to the homozygous genotypes, while the intermediate values corresponded to the heterozygous genotypes. The least-square mean (± SE) of the LWG by seven significant SNPs is shown in Table 2, which also presents the genotype and allele frequencies.

**TABLE 2:** Least square mean (± SE) of sow litter weight gain (LWG) by the genotype of seven significant SNPs.

SNP	Genotypes	*NO.*	Frequency	Allele	Frequency	Sow milk production ability (kg)
ASGA0010040	AA	260	0.2640	A	0.5020	46.8144 ± 1.1143^Aa^
	AG	469	0.4761			49.2446 ± 0.9922^b^
	GG	256	0.2599	G	0.4980	50.7648 ± 1.1816^Bb^
MARC0029355	AA	13	0.0132	A	0.1162	45.5502 ± 3.4420
	AG	203	0.2061			48.1851 ± 1.1887
	GG	769	0.7807	G	0.8838	49.0730 ± 0.9386
WU_10.2_6_65751151	AA	797	0.8091	A	0.8964	49.7458 ± 0.9377^B^
	AG	172	0.1746			45.8905 ± 1.2061^A^
	GG	16	0.0162	G	0.1036	46.1629 ± 3.1500^AB^
MARC0058875	AA	645	0.6548	A	0.7995	50.1935 ± 0.9668^B^
	AG	285	0.2893			47.1476 ± 1.0909^A^
	GG	55	0.0558	G	0.2005	44.1264 ± 1.8422^A^
WU_10.2_10_49571394	AA	8	0.0081	A	0.0736	56.2333 ± 4.3318
	AG	129	0.1310			49.9266 ± 1.3490
	GG	848	0.8609	G	0.9264	48.4391 ± 0.9337
M1GA0014659	AA	160	0.1624	A	0.3888	50.7410 ± 1.2967^b^
	AG	446	0.4528			48.6969 ± 1.0052^ab^
	GG	379	0.3848	G	0.6112	48.1426 ± 1.0410^a^
MARC0042106	AA	461	0.4680	A	0.6883	47.8839 ± 1.0019^a^
	AG	434	0.4406			49.6625 ± 1.0058^b^
	GG	90	0.0914	G	0.3117	49.3675 ± 1.5408^ab^

Note: *No.*: Number of cows with corresponding genotypes. Different letter (small letters: *p* < 0.05; capital letters: *p* < 0.01) superscripts indicate significant differences among the genotypes.

Sows that were homozygous AA for ASGA0010040 showed significantly lower LWG than those that were homozygous GG (*p* < 0.01) and heterozygous AG (*p* < 0.05). The homozygous AA for MARC0058875 showed significantly larger LWG than those with homozygous GG (*p* < 0.01) and heterozygous AG (*p* < 0.01). The homozygous AA for M1GA0014659 showed significantly larger LWG than those with homozygous GG (*p* < 0.05). Sows that were homozygous AA for WU_10.2_6_65751151 and AG for MARC0042106 showed significantly larger milk production ability than those that were heterozygous AG (*p* < 0.01) and homozygous AA (*p* < 0.05), respectively. The SNPs MARC0029355 and WU_10.2_10_49571394 were not significant, while the homozygous GG for MARC0029355 and AA for WU_10.2_10_49571394 had obvious larger LWG than those with homozygous AA and GG, respectively. These results further confirmed that the seven SNPs were highly associated with sow milk production ability.

In addition, through screening for flanking regions ± 200 kb nearby seven significant SNPs, a total of 38 genes were identified in SSCs 2, 6, 7, 10, 11, and 15 (Table 1).

### Functional Analysis of Candidate Genes

To investigate the functions of 38 genes, we performed GO and KEGG pathway analysis by KOBAS. In total, 142 GO and 51 KEGG enrichments were clustered with 28 genes (Supplementary Table S2). All these GO and KEGG enrichments were mainly related to cellular components and basic metabolism. In which, many GO and KEGG enrichments were involved in lactose metabolism, colostrum immunity, and milk protein and fat, such as tetrahydrofolate interconversion, thermogenesis, oxytocin signaling pathway, antigen processing and presentation, primary immunodeficiency, immune system process, glycoprotein catabolic process, cGMP-PKG signaling pathway, fat cell differentiation, and MAPK signaling pathway (Supplementary Table S2). Additionally, there were also many important metabolism enrichments clustered by these genes, including chloride channel activity, ubiquitin-mediated proteolysis, regulation of cell growth, carbon metabolism, metabolic pathways, ATP binding, and oxidation-reduction process (Supplementary Table S2). According to the results of the GO and KEGG enrichments, we considered the 28 genes as candidates for lactose metabolism, colostrum immunity, and milk protein and fat (Supplementary Table S2).

### Combined Analysis With the Reported Transcriptome of Swine Mammary Gland and GWAS Data of Sow Milk Production Ability

To further detect insights into the association of 28 candidate genes with milk synthesis, we performed the combined analysis between this GWAS and reported transcriptome data (Palombo et al., 2018) to improve the accuracy of the selection of functional genes related to milk production in swine. In total, nine (ANO3, MUC15, DISP3, FBXO6, CLCN6, HLA-DRA, SLA-DRB1, SLA-DQB1, and SLA-DQA1) of 28 candidates were differentially expressed genes at days 14, 10, 6, and 2 before (−) parturition and day 1 after (+) parturition (Table 3).

**TABLE 3:** Results of the combined analysis with the reported swine mammary gland transcriptome and milk production ability GWAS data.

Corresponding genes of candidate genes located in the reported QTLs						Results of the combined analysis between the previous RNA-seq and the current GWAS		
Gene	Gene symbol	QTL (bp)	Distance (bp)	Distance (Mb)	Traits (reference)	Gene	Group	*p*-value
ENSSSCG00000013351	NAV2	ssc2: 40936355	858430	0.86	LWG and EOP^b^	ANO3	(−10) vs (−14)	1.00E+00
ENSSSCG00000013338	SLC5A12	ssc2: 40936355	7322806	7.32	LWG and EOP^b^		(−6) vs (−14)	1.08E-01
ENSSSCG00000013339	ANO3	ssc2: 40936355	6949145	6.95	LWG and EOP^b^		(−2) vs (−14)	9.87E-03
ENSSSCG00000013340	MUC15	ssc2: 40936355	7237662	7.24	LWG and EOP^b^		(+1) vs (−14)	4.00E-01
ENSSSCG00000030874	NA	ssc7: 94754228	70042179	70.04	LWG^a^	MUC15	(−10) vs (−14)	1.00E+00
ENSSSCG00000030874	NA	ssc7: 118733319	94021270	94.02	LWG^a^		(−6) vs (−14)	3.81E-01
ENSSSCG00000027921	NA	ssc7: 94754228	69961637	69.96	LWG^a^		(−2) vs (−14)	2.89E-02
ENSSSCG00000027921	NA	ssc7: 118733319	93940728	93.94	LWG^a^		(+1) vs (−14)	1.49E-02
ENSSSCG00000001447	NA	ssc7: 94754228	69964042	69.96	LWG^a^	DISP3	(−10) vs (−14)	1.00E+00
ENSSSCG00000001447	NA	ssc7: 118733319	93943133	93.94	LWG^a^		(−6) vs (−14)	5.06E-01
ENSSSCG00000025071	BTNL2	ssc7: 94754228	69944374	69.94	LWG^a^		(−2) vs (−14)	1.04E-01
ENSSSCG00000025071	BTNL2	ssc7: 118733319	93923465	93.92	LWG^a^		(+1) vs (−14)	1.72E-02
ENSSSCG00000001453	HLA-DRA	ssc7: 94754228	69919068	69.92	LWG^a^	FBXO6	(−10) vs (−14)	1.00E+00
ENSSSCG00000001453	HLA-DRA	ssc7: 118733319	93898159	93.90	LWG^a^		(−6) vs (−14)	4.33E-01
ENSSSCG00000001455	SLA-DRB1	ssc7: 94754228	69840175	69.84	LWG^a^		(−2) vs (−14)	6.96E-01
ENSSSCG00000001455	SLA-DRB1	ssc7: 118733319	93819266	93.82	LWG^a^		(+1) vs (−14)	5.00E-02
ENSSSCG00000001457	SLA-DQB1	ssc7: 94754228	69776931	69.78	LWG^a^	CLCN6	(−10) vs (−14)	1.00E+00
ENSSSCG00000001457	SLA-DQB1	ssc7: 118733319	93756022	93.76	LWG^a^		(−6) vs (−14)	6.93E-01
ENSSSCG00000001456	SLA-DQA1	ssc7: 94754228	69758591	69.76	LWG^a^		(−2) vs (−14)	1.67E-01
ENSSSCG00000001456	SLA-DQA1	ssc7: 118733319	93737682	93.74	LWG^a^		(+1) vs (−14)	8.98E-02
ENSSSCG00000001459	HLA-DOB	ssc7: 94754228	69716277	69.72	LWG^a^	HLA-DRA	(−10) vs (−14)	1.00E+00
ENSSSCG00000001459	HLA-DOB	ssc7: 118733319	93695368	93.70	LWG^a^		(−6) vs (−14)	8.86E-01
ENSSSCG00000025593	TAP2	ssc7: 94754228	69697080	69.70	LWG^a^		(−2) vs (−14)	1.14E-01
ENSSSCG00000025593	TAP2	ssc7: 118733319	93676171	93.68	LWG^a^		(+1) vs (−14)	1.59E-03
ENSSSCG00000026951	PSMB8	ssc7: 94754228	69681182	69.68	LWG^a^	SLA-DQB1	(−10) vs (−14)	1.00E+00
ENSSSCG00000026951	PSMB8	ssc7: 118733319	93660273	93.66	LWG^a^		(−6) vs (−14)	6.19E-01
ENSSSCG00000001463	PSMB9	ssc7: 94754228	69675634	69.68	LWG^a^		(−2) vs (−14)	2.63E-01
ENSSSCG00000001463	PSMB9	ssc7: 118733319	93654725	93.65	LWG^a^		(+1) vs (−14)	3.72E-02
ENSSSCG00000025618	TAP1	ssc7: 94754228	69682366	69.68	LWG^a^	SLA-DQA1	(−10) vs (−14)	1.00E+00
ENSSSCG00000025618	TAP1	ssc7: 118733319	93661457	93.66	LWG^a^		(−6) vs (−14)	3.27E-01
							(−2) vs (−14)	3.18E-02
							(+1) vs (−14)	2.79E-04
						SLA-DRB1	(−10) vs (−14)	1.00E+00
							(−6) vs (−14)	6.56E-01
							(−2) vs (−14)	2.66E-01
							(+1) vs (−14)	1.63E-02

Note: GWAS: Genome-wide association study. QTL: Quantitative trait loci. NA: Novel gene in Ensembl database. (−14), (−10), (−6), (−2), and (+ 1): At days 14, 10, 6, and 2 before (−) parturition and day 1 after (+) parturition. a: The reference ∼ Thekkoot DM, Young JM, Rothschild MF, Dekkers JC: Genomewide association analysis of sow lactation performance traits in lines of Yorkshire pigs divergently selected for residual feed intake during grow-finish phase. *J Anim Sci* 2016, 94 (6):2317–2331. b: The reference ∼ Chapter 4. A genome wide association analysis for sow lactation traits in Yorkshire and Landrace sows (https://lib.dr.iastate.edu/cgi/viewcontent.cgi?article=5224&context=etd). c: The reference ∼ Palombo V, Loor JJ, D’Andrea M, Vailati-Riboni M, Shahzad K, Krogh U, Theil PK: Transcriptional profiling of swine mammary gland during the transition from colostrogenesis to lactogenesis using RNA sequencing. *Bmc Genomics* 2018, 19.

We also compared the chromosome positions of 28 candidates with those of the QTLs from reported GWAS data for milk production ability traits, and a total of 17 genes were found to be within 0.86–94.02 Mb of the reported QTLs for milk yield (Table 3). In which, NAV2 was found to be located with 0.86 Mb of QTL region ssc2: 40936355 that was confirmed to have large genetic effects on sow milk yield (Table 3).

## Discussion

In this study, we estimated the heritability and EBV of LWG and performed a GWAS to screen the candidate genes. We found 28 promising candidates involved in lactose metabolism, colostrum immunity, and milk protein and fat, such as tetrahydrofolate interconversion, primary immunodeficiency, glycoprotein catabolic process, fat cell differentiation, and MAPK signaling pathway.

Our heritability estimates for LWG were 0.18 and were consistent with those reported by DM. Thekkoot, who found the heritability of LWG ranged from 0.16 to 0.22 for Yorkshire and 0.12–0.20 for Landrace sows (Thekkoot et al., 2016a). We performed the GWAS and proposed seven significant SNPs associated with sow milk production ability. By the estimation of least-square means, ASGA0010040, MARC0058875, WU_10.2_10_49571394, M1GA0014659, and MARC0042106 were found that the extreme phenotypic values significantly corresponded to the homozygous genotypes. Sows that were genotyped for MARC0029355 and WU_10.2_10_49571394 had an obvious phenotype trend between two different homozygous, while not significant. This might be due to the high SE.

The lactation process includes initiation and maintenance, which are mainly regulated by hormone-nerve. Milk production is highly influenced by the sow’s body reserves at the start of lactation as well as the degree and type of body tissues that are mobilized during lactation (Costermans et al., 2020). Selection for high prolificacy in modern sows has led to increased litter size and a higher number of piglets weaned per litter, which results in greater metabolic demands during lactation, due to a higher milk production (Kemp et al., 2018). In our research, we found the candidate genes were enriched mainly in metabolism-related functions, especially in processes involving carbohydrates, ATP, lipids, and protein processes. In addition, we also found that these candidate genes were involved in colostrum immune processes and milk synthesis.

By the combined analysis with the swine mammary gland transcriptome data, nine genes were identified to be key candidates. By the combined analysis with the reported GWAS data, the NAV2 gene was found to be located with 0.86 Mb of the reported QTL region ssc2: 40936355. We comprehensively analyzed the results of functional enrichments, the swine mammary gland transcriptome, and previous GWAS data, which revealed that 28 candidate genes were associated with swine milk production, and 10 of them were key candidates.

For the 10 key candidate genes, NAV2 was mainly enriched into Na (+) channel (Mishra et al., 2015), nervous system development (Clagett-Dame et al., 2006; Yan et al., 2015; Pook et al., 2020), and delayed age of menopause among women (Bae et al., 2019). In all brain regions studied, the levels of NAV2 observed in late gestation and early postnatal life were the highest (Pook et al., 2020). It was reported that NAV2 was associated with hyperlipidemia (Sun et al., 2018a). ANO3 was associated with dystonia and motor neuron dysfunction (García-Hernández et al., 2021). The glycoprotein MUC15 was initially isolated from the bovine milk fat globule membrane and had a potential physiological function in signal transduction (Pallesen et al., 2008). MUC15 was involved in PI3K/AKT signaling pathway (Yue et al., 2020), and the localization of MUC15 was shown to be controlled by the ovarian hormones, oestrogen, and progesterone (Poon et al., 2014). DISP3 was a molecule between thyroid hormone and cholesterol metabolism, which used thyroid hormone to regulate serum cholesterol levels, thus participating in the metabolism and synthesis of various substances such as sugar, protein, fat, estradiol, and cortisol in the body (Zikova et al., 2009). DISP3 was also associated with the release of lipid-anchored secretory proteins (Katoh and Katoh, 2005). FBXO6 was related to ovarian cancer treatment (Ji et al., 2021) and glycoprotein quality control (Glenn et al., 2008). CLCN6 was involved in the renin-angiotensin-aldosterone system (Ji et al., 2017). SLA-DRA, SLA-DRB1, SLA-DQB1, and SLA-DQA1 were the SLA class Ⅱ genes involved in immune (Liu et al., 2015).

SLC5A12 was an active source of lactate transmembrane transporter, which is mainly involved in sodium ion transport (Martin et al., 2007; Sivaprakasam et al., 2017). FBXO2 and MAD2L2 were involved in ubiquitination processes (Li et al., 2018; Liu et al., 2021), which regulated the milk protein and fat metabolic mechanism (Liu et al., 2020a). DRAXIN was related to Akt, which could impact milk synthesis (Meli et al., 2015; Liu et al., 2020b). AGTRAP was reported to have a functional role in adipose metabolism (Ohki et al., 2017). MTHFR was involved in the metabolism of carbon, methionine, and tetrahydrofolic acid, and was related to the metabolism of milk folic acid (Page et al., 2019). MTHFR could play a role in milk protein synthesis through folic acid (Hou et al., 2015). Studies reported that MTHFR was an important candidate gene for sheep milk yield traits (Hou et al., 2015; An et al., 2016). NPPB and BTNL2 were involved in PI3K/AKT, Ca^2+^, K^+^, ATP, and immunity (Fioretti et al., 2004; Dolovcak et al., 2009; Sun et al., 2018b; Zhao et al., 2020). KIAA2013 was related to DNA methylation levels of newborns (Yeung et al., 2021). HLA-DOB, PSMB8, and TAP1 were involved in immune, protein and fat metabolism processes (Nagarajan et al., 2002; Niesporek et al., 2005; Garg, 2011; Kolbus et al., 2012; Arimochi et al., 2016; Naderi et al., 2016; Moussa et al., 2018; Yang et al., 2018; Chen et al., 2020). CACNB2 was involved in the regulation of ion membrane transport, which was related to calcium channel activity, MAPK, and oxytocin signaling pathways (Durairaj Pandian et al., 2019), and studies have shown that CACNB2 was involved in the formation of porcine marlin (Bertolini et al., 2018). NSUN6 protein might have an important function in broad aspects of embryonic development (Chi and Delgado-Olguín, 2013). KIF5C was involved in the regulation of mammalian phosphorylation (Padzik et al., 2016). As the substrate of protein kinase CK2, KIF5C cloud interacts with CK2alpha to become a negative factor of adipogenesis (Schäfer et al., 2008; Chen et al., 2017).

ENSSSCG00000030874, ENSSSCG00000027921, and ENSSSCG00000001447 genes were novel genes in the Ensembl database, while our functional analysis showed their roles in the immune system.

In conclusion, we identified seven SNPs significantly associated with sow milk production ability and propose 28 candidate genes. By integrated analysis of the biological functions, swine mammary gland transcriptome, and previous GWAS data, 10 genes (NAV2, ANO3, MUC15, DISP3, FBXO6, CLCN6, HLA-DRA, SLA-DQB1, HLA-DRB1, SLA-DQB1, and SLA-DQA1) were proposed to the key candidates. Our study provided a new insight for investigating the potential critical SNPs and genes involved in sow milk production, and the molecular information might be used to improve sow lactation performance.

## Data Availability

The datasets presented in this study can be found in online repositories. The names of the repository/repositories and accession number(s) can be found in the article/Supplementary Material.
